# Transgenically mediated shRNAs targeting conserved regions of foot-and-mouth disease virus provide heritable resistance in porcine cell lines and suckling mice

**DOI:** 10.1186/1297-9716-44-47

**Published:** 2013-07-03

**Authors:** Ye Jiao, Xiuli Gong, Junzheng Du, Mingqiu Liu, Xinbing Guo, Linlin Chen, Weinan Miao, Tao Jin, Huiyun Chang, Yitao Zeng, Zhaoxin Zheng

**Affiliations:** 1 State Key Laboratory of Genetic Engineering, Institute of Genetics, School of Life Sciences, Fudan University, Shanghai 200433, China; 2 Shanghai Laboratory of Embryo and Reproduction Engineering, Shanghai Institute of Medical Genetics, Children’s Hospital of Shanghai, Shanghai Jiao Tong University School of Medicine, Shanghai 200040, China; 3 State Key Laboratory of Veterinary Etiological Biology, National Foot-and-mouth Disease Reference Laboratory, Lanzhou Veterinary Research Institute, Chinese Academy of Agricultural Sciences, Lanzhou 730046, China

## Abstract

Foot-and-mouth disease virus (FMDV) is responsible for substantial economic losses in livestock breeding each year, and the development of new strategies is needed to overcome the limitations of existing vaccines and antiviral drugs. In this study, we evaluated the antiviral potential of transgenic porcine cells and suckling mice that simultaneously expressed two short-hairpin RNAs (shRNAs) targeting the conserved regions of the viral polymerase protein 3D and the non-structural protein 2B. First, two recombinant shRNA-expressing plasmids, PB-EN3D2B and PB-N3D2B, were constructed and the efficiency of the constructs for suppressing an artificial target was demonstrated in BHK-21 cells. We then integrated PB-EN3D2B into the genome of the porcine cell line IBRS-2 using the piggyBac transposon system, and stable monoclonal transgenic cell lines (MTCL) were selected. Of the 6 MTCL that were used in the antiviral assay, 3 exhibited significant resistance with suppressing ratios of more than 94% at 48 hours post-challenge (hpc) to both serotype O and serotype Asia 1 FMDV. MTCL IB-3D2B-6 displayed the strongest antiviral activity, which resulted in 100% inhibition of FMDV replication until 72 hpc. Moreover, the shRNA-expressing fragment of PB-N3D2B was integrated into the mouse genome by DNA microinjection to produce transgenic mice. When challenged with serotype O FMDV, the offspring of the transgenic mouse lines N3D2B-18 and N3D2B-81 exhibited higher survival rates of 19% to 27% relative to their non-transgenic littermates. The results suggest that these heritable shRNAs were able to suppress FMDV replication in the transgenic cell lines and suckling mice.

## Introduction

Foot-and-mouth disease (FMD) is a highly contagious disease that affects more than 33 species of cloven-hoofed animals, such as swine, cattle and other livestock [1]. FMD outbreaks often cause severe economic losses due to reduced productivity and the required slaughter of millions of infected or susceptible animals, and these outbreaks have even raised political disputes concerning trade embargos on animal products. Therefore, this disease is designated by the International Office of Epizootics (OIE) as a serious disease that spreads rapidly and requires socioeconomic considerations. The FMD virus (FMDV) belongs to the genus *Aphthovirus* within the family *Picornaviridae*[2]. The FMDV genome is composed of a positive-sense, single-stranded RNA molecule of approximately 8500 nucleotides and contains a unique large open reading frame.

The most commonly used strategies for controlling FMD outbreaks are routine vaccination and slaughtering of infected animals. However, livestock slaughter results in substantial economic losses. The application of inactivated and attenuated vaccines causes a risk for live or revertant viruses to escape [3,4]. RNAi is a gene silencing mechanism that has been found in all eukaryotes [5]. Double-stranded small interfering RNA (siRNA) molecules or folded short-hairpin RNA (shRNA) can induce the specific degradation of complementary messenger RNA [6]. Because of the rapidity and specificity of its effects, RNAi has been exploited as a promising technology for the control of important pathogens, including human immunodeficiency virus (HIV), herpes simplex virus (HSV) and hepatitis B virus (HBV) [7-9].

Previous studies conducted in our lab and by other groups have investigated the inhibitory effect of siRNA on FMDV replication both in vitro and in vivo [10-12]. However, like other RNA viruses, FMDV undergoes rapid mutation and is antigenically variable. Furthermore, there are seven distinguishable serological types (A, O, C, Asia 1, SAT 1, SAT 2, and SAT 3) and more than 65 subtypes [13]. Thus, one major issue in establishing RNAi as a viable approach to combat FMDV is the high genetic polymorphism exhibited by this virus. Therefore, the strategy of combining multiple siRNA/shRNA and designing shRNA that target the conserved regions of the viral genome has been suggested [14-17]. The non-structural protein genes of FMDV, such as 3D [18], 2B [16] and 3C [11], may serve as potential targets of these shRNAs.

Another crucial issue is the designing of an optimal transfer vector for the delivery of shRNA-expressing cassettes. Chen et al. [10] demonstrated that the replication-defective human adenovirus type 5 (rAd5), which expresses antiviral shRNA, significantly reduced the susceptibility of guinea pigs and swine to FMDV infection. Cong et al. [12] showed that an attenuated *Salmonella choleraesuis* (C500) vaccine strain carrying an shRNA-expressing plasmid was capable of inhibiting FMDV replication in vivo. Nevertheless, these methods are still limited because the vectors only work for a short period of time in animals. An alternative strategy is to establish a transgenic RNAi system by breeding genetically modified species. Recent developments in plants [19] and insects [20] have demonstrated that heritable RNAi can induce the resistance to some infectious diseases. In animals, Golding et al. attempted to produce transgenic cattle that expressed an shRNA that targeted the prion protein [21]. Wang et al. also reported some reduction of infectious symptoms in transgenic mice that expressed a single shRNA against FMDV [22], indicating the feasibility of this transgenic system.

In this study, we constructed recombinant plasmids that could simultaneously express two shRNAs that targeted the conserved regions of the viral polymerase protein 3D and the non-structural protein 2B. The piggyBac transposon system was used as the transgenic vector in the porcine cell line IBRS-2, and up to 100% inhibition of the FMDV replication was observed in the resulting transgenic cell lines. This antiviral capacity was effective against both serotype O and serotype Asia 1 FMDV, and it was not directly affected by the transgene copy number. In addition, after the germ-line microinjection of the linearized plasmids, the offspring of two transgenic mouse lines exhibited enhanced resistance against serotype O FMDV infection.

## Materials and methods

### Plasmids, cells, and viruses

All the experiments involving virus challenge in mice were approved by the animal ethics committee of Lanzhou Veterinary Research Institute and followed both the national guidelines for the use of animals in scientific research and the standard protocol described by the OIE.

The piggyBac transposon plasmid PB[Act-RFP]DS, PB[PGK-Neo] and the helper plasmid CMV-PBase were kindly provided by Prof. Xiaohui Wu [23]. IBRS-2 cells (swine kidney cells) and all of the IBRS-2-derived transgenic cell lines were cultured in Dulbecco’s modified Eagle’s medium (DMEM, Gibco, Carlsbad, USA) supplemented with 5% heat-inactivated fetal bovine serum (FBS) and adjusted to pH 7.4. BHK-21 cells (hamster kidney cells) were also cultured in DMEM but with 10% FBS. The cultures were incubated at 37°C with 5% CO_2_. FMDV isolates of serotype O/HKN/2002 [GenBank: AY317098] and serotype Asia 1/Jiangsu/2005 [GenBank: EF149009] were used for the viral challenge experiments.

### Construction of shRNA-coding vectors

The 3D-shRNA-expressing cassette was modified from the pPOL plasmid, a construct previously generated by our lab [10]. A revised mouse U6 promoter (mU6) was PCR-amplified and cloned into pPOL, replacing the original U6 promoter. The 56-bp 3D-specific shRNA-coding region inserted after the mU6 promoter was also modified to 5’-GAGGCCATCCTCTCCTTTGCACGCCGTGGGACCATACAGGAGAAGTTGATCTCCGT-3’ (sense, corresponding to nt 1225 to 1280 of O/HKN/2002 3D). Finally, the *Hind*III site after the transcriptional terminal signal of five T residues was excised by Klenow (large fragment) treatment and blunt end ligation. A lacZ-shRNA-expressing cassette was constructed in the same manner using pLacZ [10] as a control for non-specific RNAi.

The 2B-shRNA-expressing cassette was driven by a human H1 promoter (hH1), which was cloned by PCR amplification from human genomic DNA extracted from 293 T cells with the Axygen Genomic DNA Kit (Corning, Tewksbury, USA). Inverted repeats representing the 2B-shRNA or the heterologous NTH21-shRNA were inserted after the H1 promoter along with the type III RNA polymerase terminal signal of the five T residues. The 25-bp 2B-specific shRNA was 5’-CCAGATGCAGGAGGATATGTCAACA-3’ (sense, corresponding to nt 57 to 81 of O/HKN/2002 2AB), which was also rearranged based on previous work performed by our lab [12]. The 21-bp sequence of the heterologous NTH21-shRNA has been described previously [24].

The reporter gene cassette of EGFP was obtained by cloning the mouse phosphoglycerate kinase (PGK) promoter into pEGFP-N1, replacing the CMV promoter. The resulting pPGK-EGFP-N1 plasmid was digested with *Apa*I and *Afl*II. The neo-resistance (Neo-R) cassette was PCR amplified from pEGFP-N1 along with the designated terminal restriction enzyme sites.

**Figure 1:**
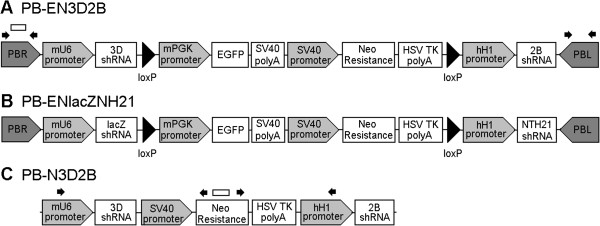
**Schematic diagram of the shRNA-expressing vectors used for transgenic modification.** (**A**) PB-EN3D2B, with a tandem array of cassettes that express the 3D-shRNA, EGFP, Neo-R, and the 2B-shRNA between the left and right transposon arms of the piggyBac transposon. Two loxP sites flank the reporter genes. (**B**) PB-ENLacZNH21, is identical to PB-EN3D2B except that the two FMDV-targeting shRNA cassettes have been substituted with non-specific shRNA cassettes. (**C**) PB-N3D2B, contains the fragment of two antiviral shRNA cassettes and the Neo-R reporter gene, which can be digested from the transposon vector for germ-line transgene; the transposon arms are not shown. The pairs of small black arrows indicate the primers used to detect the transgenic cell lines or mice. White bars indicate the PCR-amplified fragments used to determine the transgene copy number.

Combinations of the above shRNA-coding cassettes and reporter gene cassettes were cloned into the *Asc*I-*Hind*III sites between the transposon arms of the plasmid PB[Act-RFP]DS to obtain the following plasmid vectors for transgenic modification: PB-EGFP-Neo-3DshRNA-2BshRNA (PB-EN3D2B, 1A); PB-EGFP-Neo-lacZshRNA-NTH21shRNA (PB-ENlacZNH21, Figure 1B), which is a heterologous RNA-interference control vector; and PB-Neo-3DshRNA-2BshRNA (PB-N3D2B, Figure 1C). The first two vectors each contained two loxP sites at the outer edges of their reporter gene cassettes and were used for cultured cell modification, whereas PB-N3D2B was used for the preparation of transgenic mice.

### Analysis of the repression effects induced by shRNA-coding vectors against target EGFP expression in BHK-21 cells

A DNA fragment corresponding to nt 1204 to 1290 of O/HKN/2002 3D and nt 37 to 92 of O/HKN/2002 2AB in tandem was artificially synthesized and inserted into the *Sal*I-*BamH*I site of pEGFP-N1 to form the fusion protein reporter plasmid pEGFP-Target. To analyze the suppressing effects of the shRNA-coding vectors against the reporter plasmid, 0.8 μg of pEGFP-Target and 0.2/0.4/0.8/0 μg of PB-N3D2B were co-transfected using Lipofectamine 2000 (Invitrogen, Carlsbad, USA) into the monolayers of BHK-21 cells in 12-well plates. All of the transfection systems were supplemented up to 1.6 μg of DNA with a piggyBac backbone plasmid PB[PGK-Neo]. After 48 h, the cells were harvested and total RNA was isolated with an RNeasy Mini Kit (Qiagen, Hilden, Germany) and then treated with RNase-free DNase for one hour. The cDNA samples were synthesized by reverse transcription with Oligo-dT (Takara, Dalian, China) and then subjected to quantitative PCR (qPCR). The fusion GFP expression levels were detected using the primers listed in Table 1, with β-actin as the reference gene.

**Table 1:** The PCR primers used for detection and qPCR of transgenic cells and mice

**Name**	**Sequence**	**Function**
GFP-F	GGTCTTGTAGTTGCCGTCGT	Quantitate GFP in BHK-21 cells
GFP-R	TCGTGACCACCCTGACCTAC	
h-actin-F	CACGCAGCTCGTTGTAGAAG	Quantitate β-actin in BHK-21 cells
h-actin-R	AGGACTCGTACGTGGGTGAC	
PBL1-F	CAACATACGAGCCGGAAGCATAAA	Detect PBL in cell lines
PBL1-R	TAACAGAATCTTGACCTTGCCACA	
PBR1-F	AGTCAGTCAGAAACAACTTTGGCACA	Detect PBR in cell lines
PBR1-R	ACGAATAGGTGGCCTATGGCATTATTG	
PBase-F	GCCACCATGGGATGTTCTTTAG	Detect transposase in cell lines
PBase-R	GTACTCAGAAACAACTTTGGC	
Q-PBR1-F	TACGCATAAACGATGACG	Quantitate copy number in cell lines
Q-PBR1-R	CAAACTAAAGGCGGAGTG	
Neo-2B-F	TGACCGCTTCCTCGTGCTTT	Detect transgenic mice
Neo-2B-R	TGGGTGTTCCCGCCTAGTGA	
3D-Neo-F	ATCCGACGCCGCCATCTCTA	Detect transgenic mice
3D-Neo-R	CGGAGAACCTGCGTGCAATC	
NR-F	CGTTTCGCATGATTGAACAAGAT	Quantitate copy number in mice
NR-R	GTGCCCAGTCATAGCCGAAT	
GI-F	GGTGGTGAATACCATGTACAAAGCT	Quantitate GAPDH as reference in mice
GI-R	CCAACACCCCCAGTCATACG	

### Preparation and identification of monoclonal transgenic cell lines

IBRS-2 cells were seeded in 24-well plates, and 0.2 μg of plasmid PB-EN3D2B (or PB-ENlacZNH21) together with 0.2 μg of the transposase-expressing helper plasmid CMV-PBase were co-transfected into each well using Lipofectamine 2000. After 24 h of incubation, the cells were digested and approximately 1/20 to 1/40 of the cells in a single well were added to a 10-cm plate containing DMEM and 5% FBS. On the following day, the medium was replaced with DMEM containing 5% FBS and 300 μg/mL G418 (Sigma-Aldrich, St.Louis, USA). After 12–14 days of incubation, monoclonal colonies were individually collected with clone cylinders and transferred to 48-well plates. The genomic DNA of each monoclonal cell line was extracted using an Axygen Genomic DNA Kit. The primer pairs PBL1F/PBL1R and PBR1F/PBR1R, which amplify specific fragments in the left and right transposon arms, respectively, were used to identify the transgenic cell lines (Table 1 and Figure 1A). PCR tests using the primers PBase-F and PBase-R were performed at the same time to ensure that the gene encoding transposase had not been inserted. The resulting MTCL generated from the plasmids PB-EN3D2B and PB-ENlacZNH21 were named IB-3D2B and IB-lacZNH, respectively.

To detect the transgene copy numbers in the MTCL, genomic DNA samples were used as templates for qPCR with the primers Q-PBR1-F and Q-PBR1-R to amplify a 229-bp fragment of the right transposon arm (PBR). The results were normalized to the absolute amount of DNA in each sample.

### Production and selection of transgenic mice

For germ-line transgenesis, female Kunming White (KM) mice that had been pretreated with PMSG (pregnant mare serum gonadotropin) and HCG (human chorionic gonadotropin, Sigma-Aldrich) were mated with male KM mice as fertilized egg donors. The 2.5-kb *Asc*I-*Hind*III fragment of the plasmid PB-N3D2B (Figure 1C) was purified and diluted to a concentration of 2 ng/μL. After the male pronucleus was microinjected with the DNA solution, the eggs were transferred into the oviducts of pseudopregnant KM females to generate transgenic mice.

Tail tips (approximately 1-cm long) were collected from all of the mice offspring at 3 weeks of age and incubated at 56°C in a buffer of 50 mM Tris (pH 8.0), 100 mM EDTA, 100 mM NaCl, 1% SDS and 0.5 mg/mL protease K. After 3 h, the mouse genomic DNA was isolated by phenol-chloroform extraction and ethanol precipitation. The DNA samples were then dissolved in TE (pH 8.0), and the transgenic status of the mice was examined using PCR amplification with the primer pairs 3D-Neo-F/3D-Neo-R and Neo-2B-F/Neo-2B-R (Table 1 and Figure 1C), which could detect the presence of the 3D and 2B-shRNA cassettes. The transgene copy numbers in transgenic mice were examined by qPCR using the corresponding primers and with GAPDH as the reference gene. All transgenic founder mice were crossed with wild-type (WT) KM mice to maintain the descendants as hemizygotes.

### Virus challenge in transgenic cell lines

The 50% tissue culture infective doses (TCID_50_) of FMDV were calculated using the Reed-Muench formula [25]. Viral suspensions of 10^6^ to 10^7^ TCID_50_/mL were used for the experiments. To assess the antiviral capacity of the MTCL, each of the cell lines was seeded in 96-well plates. On the following day, 20 or 50 TCID_50_ of FMDV diluted in 0.1 mL DMEM without FBS were added to the monolayers. After one hour of absorption, the virus dilutions were removed, and the cells were washed with DMEM and then incubated at 37°C with 5% CO_2_. The cytopathic effects (CPE) were examined microscopically over 3 days. Supernatant fluids were harvested at various times after infection, and viral titers (i.e., TCID_50_) at 24-h intervals were determined three times in BHK-21 cells.

### Virus challenge in transgenic suckling mice

Suckling mice (2 to 3 days old) were inoculated with four 10-fold serially diluted virus solutions (i.e., 10^-6^, 10^-7^, 10^-8^, and 10^-9^) in phosphate-buffered saline (PBS) by subcutaneous injection in the neck area. The 50% lethal dose (LD_50_) was estimated using the Reed-Muench method [25]. To investigate the antiviral capacities of the transgenic mice, hemizygous male mice from transgenic mouse lines were crossed with WT female KM mice, and the newborn offspring (with a transgenic ratio of approximately 50%) were challenged with different doses of FMDV in 0.2 mL of PBS by subcutaneous injection. Tail samples were collected from every suckling mouse one day before the virus challenge. After the 5 days of observation, the genomic DNA extraction and transgenic-status differentiation were performed as described above. The survival rate of the transgenic mice was recorded, and their non-transgenic littermates were used as controls.

### Statistical analysis

GraphPad Prism 5.0 software was used for all the statistical analyses.

## Results

### The shRNA-coding vectors effectively suppressed target fusion GFP protein expression in BHK-21 cells

**Figure 2:**
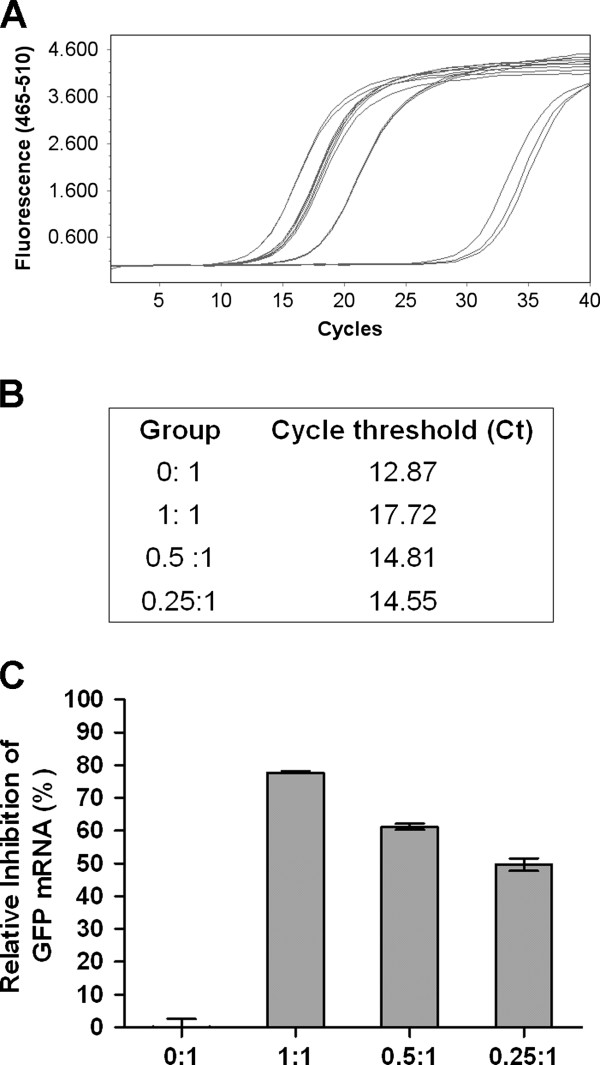
**The shRNA-coding plasmid can inhibit the artificial target gene in BHK-21 cells.** The shRNA-coding plasmid PB-N3D2B was co-transfected with the fusion GFP-expressing plasmid, pEFGP-Target, at different ratios (0:1, 1:1, 0.5:1, and 0.25:1). The mRNA levels of GFP were measured by qPCR. (**A**) One amplification plot of three separate experiments is shown. The y-axis represents the PCR relative fluorescence units at the wavelength of 465 to 510 nm. The cycle number is displayed on the x-axis. (**B**) Cycle threshold (CT) values are derived from the amplification profiles shown in panel **A**. (**C**) The suppression ratio of different amounts of PB-N3D2B compared to the control with no PB-N3D2B (transfection ratio 0:1) is shown. The data represent the means ± SEM of three separate experiments.

To identify the interfering effect of the shRNA-coding vectors, plasmid PB-N3D2B was co-transfected with pEGFP-Target into BHK-21 cells at different mass ratios of 0:1, 1:1, 0.5:1, and 0.25:1. The GFP expression of each sample was determined by qPCR. Figure 2 shows that when the two plasmids were co-transfected in the mass ratio of 1:1, PB-N3D2B was able to inhibit 77.7% of the artificial target mRNA. As the amount of PB-N3D2B decreased, the suppression efficiencies of the target mRNA were 61.2%, and 49.7%, respectively. This response occurred in a dose-dependent manner compared to the control with no PB-N3D2B (transfection ratio of 0:1).

### Establishment of shRNA-expressing monoclonal transgenic cell lines

**Figure 3:**
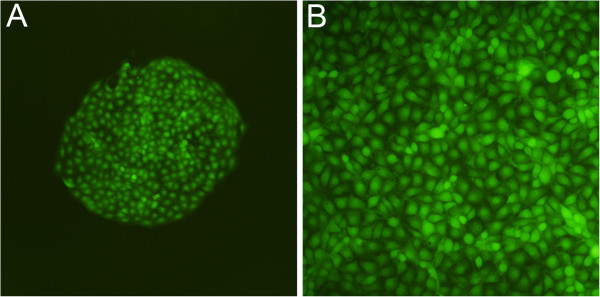
**Establishment of transgenic monoclonal colonies and the development of MTCL.** The shRNA-expressing plasmids PB-EN3D2B or PB-ENlacZNH21 were integrated into the genome of IBRS-2 cells using the piggyBac transposon system. Monoclonal colonies with both G418 resistance and EGFP expression were selected (**A**, magnification is 100×). The colonies were then cultured to generate cell lines. All of the cells expressed EGFP (**B**, magnification is 200×). The images were adjusted for contrast and dpi (dot per inch) to improve their appearance.

The PB-EN3D2B and PB-ENlacZNH21 plasmid were integrated into the genome of IBRS-2 cell using the piggyBac transposon system. Monoclonal colonies with both G418 resistance and EGFP expression (Figure 3A) were harvested and developed into monoclonal transgenic cell lines (Figure 3B). By PCR confirmation, we obtained 16 IB-3D2B and 3 IB-lacZNH MTCL. After monoclonal selection, all cell lines were passaged without G418 at least 10 times before the virus challenges.

### The shRNA genes conferred resistance against serotype O FMDV infection in transgenic cell lines

**Figure 4:**
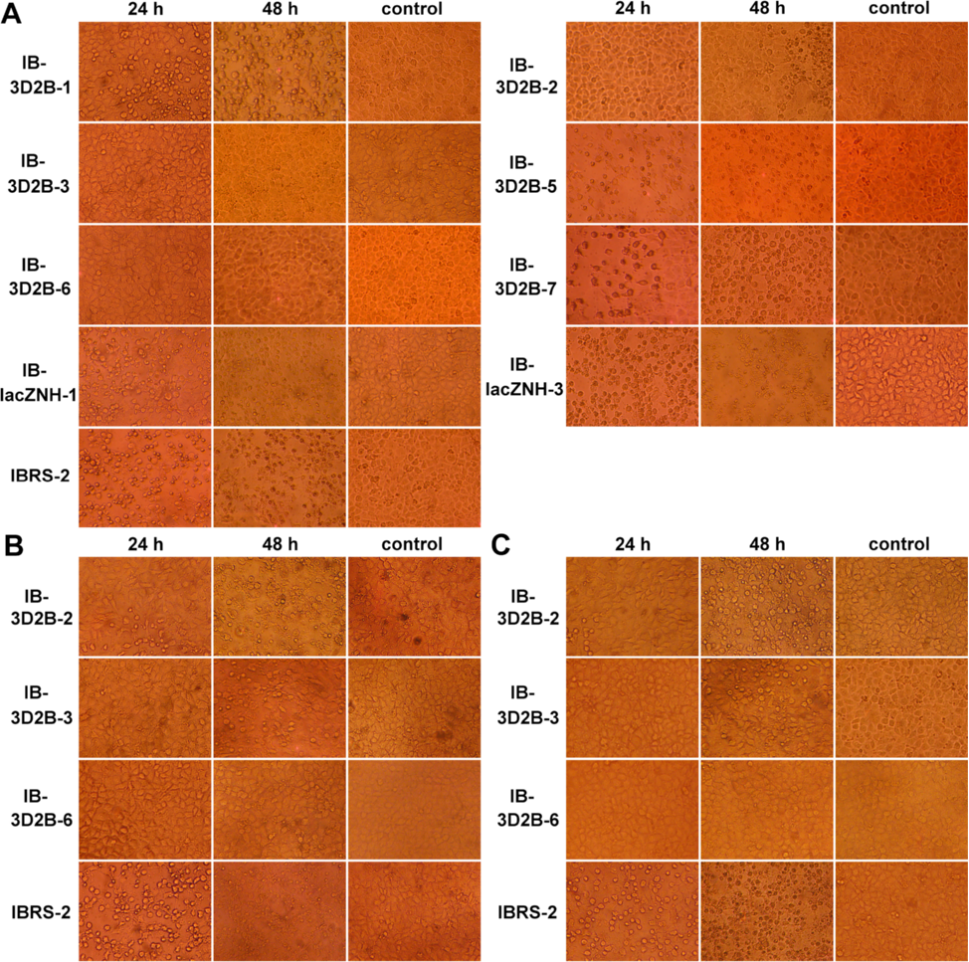
**Endogenous shRNA in MTCL conferred natural resistance to FMDV infection.** Eight cell lines and normal IBRS-2 cells were challenged with 20 TCID_50_ of FMDV O/HKN/2002 (**A**), 50 TCID_50_ of FMDV O/HKN/2002 (**B**) or 20 TCID_50_ of FMDV Asia 1/Jiangsu/2005 (**C**). At 24 hpc (left column) and 48 hpc (middle column), cells were observed under a microscope. Mock challenged cells from the same cell lines at 48 hpc were used as controls (right column). Normal IBRS-2 cells form an epithelial-like monolayer. These cells round up, separate, and finally undergo cell lysis (CPE) when infected by FMDV. The MTCL IB32-2 and IB32-3 exhibited significant antiviral capacity with considerable delayed CPE. IB32-6 had the strongest anti-FMDV capacity and was undamaged by all three challenges. The images were adjusted for contrast and dpi to improve their appearance, and the magnification for all images is 200 ×.

**Figure 5:**
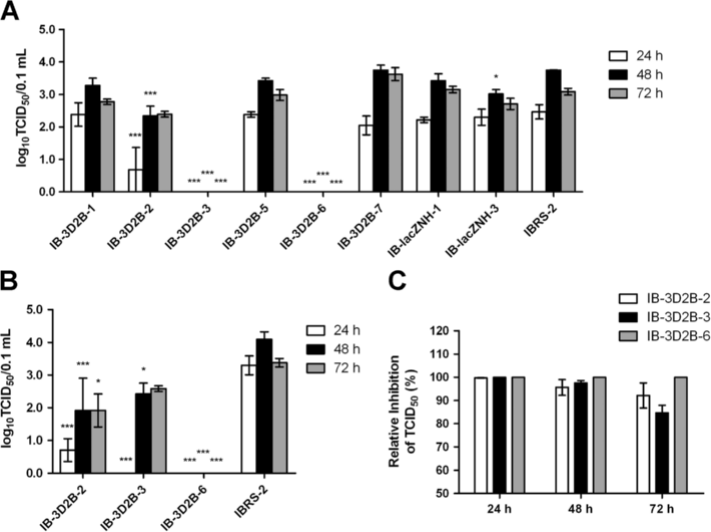
**Endogenous shRNA in transgenic cell lines conferred natural resistance to serotype O FMDV infection.** Eight cell lines and control cells were challenged with 20 TCID_50_ of FMDV O/HKN/2002. Culture supernatants were collected at 24, 48 and 72 hpc, and virus yields were measured by TCID_50_ (**A**). Three of the cell lines with an obvious antiviral ability were further challenged with a higher dose of 50 TCID_50_ of serotype O virus. Supernatant virus titers (**B**) and the relative titer reduction (**C**) were measured. The data represent the means ± SEM of three separate experiments. Asterisks indicate the samples that are different from normal IBRS-2 cells at the same time interval as determined by two-way ANOVA with Bonferroni post-test, *P < 0.05, ***P < 0.001.

To evaluate whether the presence of transgenic shRNA genes correlated with antiviral activity, 6 strains of IB-3D2B were infected with 20 TCID_50_ of FMDV O/HKN/2002. Two strains of IB-lacZNH and normal IBRS-2 cells were submitted to virus challenge at the same time as controls. The cells were observed every 24 h under a microscope. Figure 4A shows that all the control cells, including normal IBRS-2 and the two heterologous shRNA-expressing IB-lacZNH-1 and IB-lacZNH-3 strains, exhibited extensive CPE within 24 hpc. The IB-3D2B-1, IB-3D2B-5, and IB-3D2B-7 cells showed slight CPE at 24 hpc, but they exhibited severe CPE after 48 hpc. However, the cell line IB-3D2B-2 showed significant resistance to FMDV with only mild CPE at 48 hpc. The cell lines IB-3D2B-3 and IB-3D2B-6 had the strongest antiviral capacities because no CPE was observed in these cells after 3 days of observation. We also investigated the inhibitory effect of endogenous shRNA on virus replication by measuring the viral titers in the supernatants of each cell culture at 24-h intervals. These results were consistent with the microscopic observations (Figure 5A). No FMDV was detected in the supernatants of the IB-3D2B-3 and IB-3D2B-6 cells. The supernatant viral titers of the IB-3D2B-2 cells were reduced by 90% at 24 hpc and by 94% at 48 hpc compared to the non-transgenic IBRS-2 cells (*P* < 0.001). The other three transgenic cell lines, IB-3D2B-1, IB-3D2B-5, and IB-3D2B-7, and the two control cell lines, IB-lacZNH-1 and IB-lacZNH-3, exhibited no significant differences compared with the normal IBRS-2 cells.

To further study the antiviral potential of the MTCL, the three transgenic cell lines IB-3D2B-2, IB-3D2B-3, and IB-3D2B-6 were challenged with 50 TCID_50_ of FMDV O/HKN/2002, and normal IBRS-2 cells were used as controls. Microscopic examination revealed that there was an obvious delay in the CPE development in IB-3D2B-2 and IB-3D2B-3 cells challenged with 50 TCID_50_ of virus, whereas the IB-3D2B-6 cells, which had stronger antiviral activity, failed to develop any CPE after 3 days of observation (Figure 4B). The virus was not detectable in the supernatant fluid of IB-3D2B-6 cells. IB-3D2B-2 and IB-3D2B-3 also showed significantly reduced titers within 48 hpc (at least 95% of reduction, Figure 5B and C).

### The shRNA genes provided inhibitory effects against serotype Asia 1 FMDV in the transgenic cell lines

**Figure 6:**
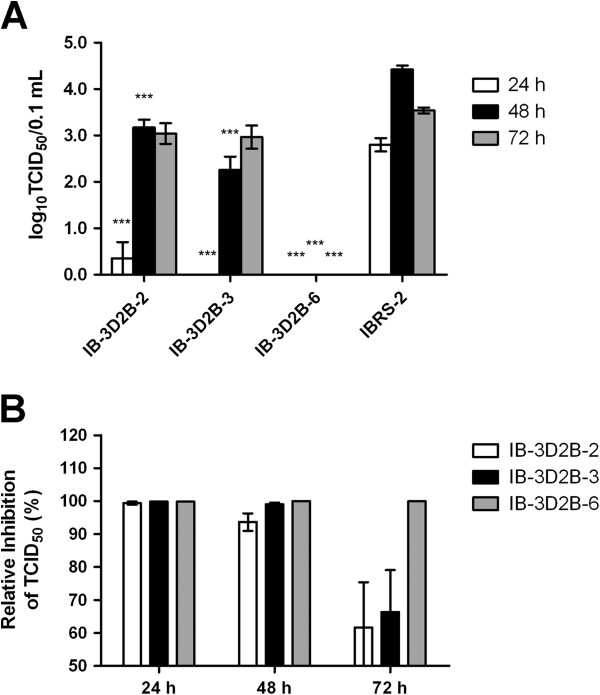
**Endogenous shRNA in transgenic cell lines provided an inhibitory effect to serotype Asia 1 FMDV infection.** Three cell lines, IB-3D2B-2, IB-3D2B-3, and IB-3D2B-6, were challenged with 20 TCID_50_ of FMDV Asia 1/Jiangsu/2005. Supernatant virus titers (**A**) and the relative titer reduction (**B**) were measured. The data represent the means ± SEM of three separate experiments. Asterisks indicate the samples that are different from normal IBRS-2 cells at the same time interval as determined by two-way ANOVA with Bonferroni post-test, *P < 0.05, ***P < 0.001.

The shRNAs that we applied in this investigation targeted the conserved regions of the FMDV genome, which theoretically enabled cross-inhibition of infection by different FMDV serotypes. To test this capacity, the MTCL of IB-3D2B-2, IB-3D2B-3, and IB-3D2B-6 were challenged with 20 TCID_50_ of FMDV Asia 1/Jiangsu/2005. As shown in Figure 4C and Figure 6, delayed CPE and reduced supernatant virus titers were found in cell lines IB-3D2B-2 and IB-3D2B-3. Again, IB-3D2B-6 completely inhibited viral replication. Thus, the antiviral ability of the selected cell lines was effective against both serotype O and serotype Asia 1 FMDV.

### The shRNA gene copy number does not directly affect the degree of resistance in the transgenic cell lines

Because the piggyBac transposon system appeared to randomly integrate DNA fragments into the genome at multiple sites, we attempted to analyze the correlation between the antiviral effects and the individual transgenic statuses of the MTCL. The shRNA gene copy numbers of the 8 MTCL were determined by qPCR, and the results are listed in Table 2. The IB-3D2B-3 cells that had the highest copy number (13 integrations) did strongly inhibit FMDV replication; however, the IB-3D2B-1 cells with a copy number of 11 did not display significant inhibitory effect against FMDV. Likewise, the IB-3D2B-2 and IB-3D2B-5 cells had similar copy numbers, but their antiviral capacities were quite different. Furthermore, the IB-3D2B-6 cells that had the highest anti-FMDV capacity contained only one copy of the shRNA-expressing genes.

**Table 2:** Correlation between transgene copy numbers and antiviral capacities of the MTCL.

**Cell line**	**Copy number**	**Antiviral capacity**
IB-3D2B-1	11	-^a^
IB-3D2B-2	5	+
IB-3D2B-3	13	+
IB-3D2B-5	6	-
IB-3D2B-6	1	+++
IB-3D2B-7	3	-
IB-lacZNH-1	7-8	-
IB-lacZNH-3	13	-

a: Semi-quantitative analysis of the antiviral capacities: - is negative and similar to non-transgenic cells; +++ is maximum.

### Anti-FMDV shRNA enhanced the survival rates of transgenic suckling mice after virus challenge

Two lines of transgenic mice, N3D2B-18 (copy number = 1) and N3D2B-81 (copy number = 2), were used for in vivo studies to assess the potential anti-FMDV activity of endogenous shRNA. The suckling mice from the two lines were first challenged with a relatively high dose of 10 LD_50_ FMDV O/HKN/2002. Neither of the transgenic lines exhibited significant resistance to FMDV infection (Table 3). To further evaluate the antiviral capacities of these transgenic mice, the challenge dosage was reduced to 3 and 6 LD_50_. The results are illustrated in Table 3. When challenged with 3 LD_50_ of type O FMDV, 7 out of the 26 transgenic suckling mice (26.9%) from line N3D2B-18 survived after all 27 non-transgenic littermates had died (*P* < 0.01). In the line N3D2B-81 mice, the survival rate at this dosage increased from 22.2% in the littermates to 41.2% in the transgenic mice. Using a challenge dose of 6 LD_50_, transgenic suckling mice from line N3D2B-18 showed a slight increase (14.3% compared to 9.1%) in the survival rate compared with controls. However, the N3D2B-81 mice showed greater antiviral capacities at this dosage, and the rate of survival was 27.3% compared to 0% for the littermates.

**Table 3:** Survival rates of suckling mice from different transgenic mouse lines challenged with serotype O FMDV

**Mouse line**	**Dosage(LD**_**50**_**)**	**Transgenic suckling mice** **Survival/Total (%)**	**Non-transgenic littermates** **Survival/Total (%)**
N3D2B-18	10	0/9 (0)	1/16 (6.3)
	6	3/21 (14.3)	3/33 (9.1)
	3	7/26 (26.9)	0/27 (0)
N3D2B-81	10	0/9 (0)	0/22 (0)
	6	3/11 (27.3)	0/23 (0)
	3	7/17 (41.2)	2/9 (22.2)

## Discussion

Although the technique for producing transgenic animals that express shRNA was first reported in 2002 [26-28], few studies have explored the prevention of virus replication in mammals using this strategy. Wang et al. attempted to generate a transgenic mouse model containing a single anti-FMDV shRNA gene [22]. Immunohistochemical assays demonstrated a reduction of the virus in the tissues of infected adult primary transgenic mice. Another report by Wang and Wu discussed the antiviral potential of shRNA-expressing cells and primary cells from transgenic bovine fetuses made by lenti-virus insertion [29]. However, no reports have evaluated whether the transgenic FMDV-susceptible animals and their transgenic offspring were capable of resisting to viral infection. In this study, we constructed plasmids that simultaneously encoded two shRNAs that targeted conserved regions of the FMDV genome. The results demonstrated that the plasmid PB-N3D2B was able to effectively suppress an artificial target in vitro, and transgenic modification with these plasmids provided strong antiviral capacity in repeatedly passaged cell lines and enhanced resistance against FMDV infection in offspring suckling mice. Our present experimental data suggest that transgenic RNAi is worthy of pursuit as a strategy for producing animals resistant to FMDV.

The method of combining multiple conserved region-targeting siRNA/shRNA has been the preferred antiviral strategy because one shRNA can complement the other, with the added benefit of potentially suppressing mutated or heterologous viruses [16,30]. Notably, there is evidence that the co-expression of multiple shRNAs from a single construct appears to be more effective than simply mixing single shRNAs [17]. In this investigation, we simultaneously expressed a 25-bp 2B-targeting and a 56-bp 3D-targeting shRNA. The 2B gene is involved in membrane rearrangements that are required for viral RNA replication and capsid assembly [31]. The 3D gene encodes the RNA-dependent RNA polymerase, the fundamental protein that conducts viral genome replication [32]. We did not employ a typical 19-bp shRNA because the 56- and 25-bp shRNA-corresponding sequences in different serotypes of FMDV were highly conserved, and these longer shRNAs had strong anti-FMDV effects [10,12]. This phenomenon may be attributed to the potential diversity of the siRNA products made from long shRNAs by Dicer, which mimics the combination of multiple shRNAs.

The short half-life of siRNA/shRNA has always been a challenge for in vivo tests. In most reports, RNAi treatment might be effective only if the injection of siRNA/shRNA was performed within two days before the virus inoculation. In particular, the commonly used RNAi delivery system, recombinant adenovirus (rAd), has unavoidable shortcomings. The rAd-derived non-coding RNA VA1 can be expressed so strongly that it saturates the exportin-5 pathway, which is necessary for the transport of both foreign shRNA and cellular microRNA [33,34]. The differential tissue distributions of rAd and FMDV are an additional complication [35]. In this study, the MTCL were subjected to two rounds of monoclonal selection and were passaged at a 1:2 to 1:3 ratio 10 to 15 times before virus challenge, and F3 or F4 transgenic suckling mice underwent antiviral testing. Due to the genomic stability of our transgenic products, transgenic animals express natural resistance and do not require on-going precautionary inoculations.

The position effect is a major factor that determines transgene expression levels [36]. Specifically, the expression of foreign genes is influenced by the chromosome structure surrounding the integration sites and cis-acting elements, such as enhancers and insulators. This effect makes the expression of transgenes unpredictable. Although we used polIII promoters (U6 and H1) instead of the polII promoter (for instance, CMV), which is usually used for foreign protein expression, a similar phenomenon was observed in our experiments. Assuming that the RNA expression level is positively related to the antiviral effect, the capacity of suppressing FMDV is the accumulation of all the integrating sites in the transgenic cell lines. As for the microinjection method in mouse experiments, however, foreign gene fragments tend to integrate into a few sites but with multiple copies. Once the fate of the entire transgene cluster is determined by its local position, the copy number and homozygous/hemizygous status do affect gene expression [37,38]. In this study, we selected low-copy and hemizygous transgenic lines to avoid possible genetic side effects, such as chromosomal recombination and lethal homozygosity. This selection may partly explain the relatively limited antiviral capacities of the transgenic mouse lines. As primary cell selection and nuclear transfer is the principle technique of breeding large transgenic animals, locus control regions (LCR) [39] or artificial chromosomes [40] could be employed as position-independent delivery methods to elicit more efficient RNAi expression and to reduce the cost of selecting effective offspring.

In conclusion, we show that heritable FMDV resistance can be conferred by two endogenous shRNAs in susceptible cells and animals. To further improve this strategy, we are currently developing a multiple conserved region-targeting shRNA gene to confer strong, broad-spectrum resistance to FMDV infections of different serotypes. Additionally, novel transgenic methods in combination with conditional RNAi induction are expected to prevent side effects in livestock.

## Competing interests

The authors declare that they have no competing interests.

## Authors’ contributions

YJ: constructed the recombinant plasmid, cultured and selected the transgenic cell lines, carried out the virus challenge assays on cell lines and mice, and drafted the manuscript. XLG: microinjected the DNA to fertilized eggs and breed the transgenic mice. JZD: carried out the virus challenge on mice. MQL: participated in designing the whole experiment and revised the manuscript critically. XBG: carried out the PCR determination of the transgenic mice. LLC: participated in culturing transgenic cell lines and the virus challenge on mice. WM: participated in the virus challenge on mice. TJ: carried out the qPCR test on BHK-21 cells. HYC: participated in designing the experiment of virus challenge. YTZ: participated in designing the experiment of breeding transgenic mice. ZXZ: participated in designing the whole experiment and revised the manuscript critically. All authors read and approved the final manuscript.

## References

[B1] Pereira HG Gibbs EPJ Foot-and-mouth disease Virus Diseases of Food Animals 1981 London: Academic Press Inc 333 363

[B2] Goodwin EC Rottman FM The 3'-flanking sequence of the bovine growth-hormone gene contains novel elements required for efficient and accurate polyadenylation J Biol Chem 1992 267 16330 16334 1644817

[B3] King AMQ Underwood BO McCahon D Newman JWI Brown F Biological identification of viruses causing the 1981 outbreaks of foot and mouth disease in the UK Nature 1981 293 479 480 10.1038/293479a0 6273731

[B4] Barteling SJ Vreeswijk J Developments in foot-and-mouth-disease vaccines Vaccine 1991 9 75 88 10.1016/0264-410X(91)90261-4 1647575

[B5] Fire A RNA-triggered gene silencing Trends Genet 1999 15 358 363 10.1016/S0168-9525(99)01818-1 10461204

[B6] Caplen NJ Parrish S Imani F Fire A Morgan RA Specific inhibition of gene expression by small double-stranded RNAs in invertebrate and vertebrate systems Proc Natl Acad Sci USA 2001 98 9742 9747 10.1073/pnas.171251798 11481446 PMC55523

[B7] Hamasaki K Nakao K Matsumoto K Ichikawa T Ishikawa H Eguchi K Short interfering RNA-directed inhibition of hepatitis B virus replication FEBS Lett 2003 543 51 54 10.1016/S0014-5793(03)00400-9 12753904

[B8] Palliser D Chowdhury D Wang QY Lee SJ Bronson RT Knipe DM Lieberman J An siRNA-based microbicide protects mice from lethal herpes simplex virus 2 infection Nature 2006 439 89 94 10.1038/nature04263 16306938

[B9] Jacque JM Triques K Stevenson M Modulation of HIV-1 replication by RNA interference Nature 2002 418 435 438 10.1038/nature00896 12087358 PMC9524216

[B10] Chen W Liu M Jiao Y Yan W Wei X Chen J Fei L Liu Y Zuo X Yang F Lu Y Zheng Z Adenovirus-mediated RNA interference against foot-and-mouth disease virus infection both in vitro and in vivo J Virol 2006 80 3559 3566 10.1128/JVI.80.7.3559-3566.2006 16537624 PMC1440392

[B11] Kim SM Lee KN Park JY Ko YJ Joo YS Kim HS Park JH Therapeutic application of RNA interference against foot-and-mouth disease virus in vitro and in vivo Antiviral Res 2008 80 178 184 10.1016/j.antiviral.2008.06.001 18601955

[B12] Cong W Jin H Jiang C Yan W Liu M Chen J Zuo X Zheng Z Attenuated salmonella choleraesuis-mediated RNAi targeted to conserved regions against foot-and-mouth disease virus in guinea pigs and swine Vet Res 2010 41 30 10.1051/vetres/2010002 20167192 PMC2826090

[B13] Beck E Strohmaier K Subtyping of European foot-and-mouth-disease virus strains by nucleotide sequence determination J Virol 1987 61 1621 1629 3033288 10.1128/jvi.61.5.1621-1629.1987 PMC254144

[B14] Gitlin L Andino R Nucleic acid-based immune system: the antiviral potential of mammalian RNA silencing J Virol 2003 77 7159 7165 10.1128/JVI.77.13.7159-7165.2003 12805414 PMC164787

[B15] Song EW Lee SK Dykxhoorn DM Novina C Zhang D Crawford K Cerny J Sharp PA Lieberman J Manjunath N Shankar P Sustained small interfering RNA-mediated human immunodeficiency virus type 1 inhibition in primary macrophages J Virol 2003 77 7174 7181 10.1128/JVI.77.13.7174-7181.2003 12805416 PMC164789

[B16] Kahana R Kuznetzova L Rogel A Shemesh M Hai D Yadin H Stram Y Inhibition of foot-and-mouth disease virus replication by small interfering RNA J Gen Virol 2004 85 3213 3217 10.1099/vir.0.80133-0 15483234

[B17] Kim SM Lee KN Lee SJ Ko YJ Lee HS Kweon CH Kim HS Park JH Multiple shRNAs driven by U6 and CMV promoter enhances efficiency of antiviral effects against foot-and-mouth disease virus Antiviral Res 2010 87 307 317 10.1016/j.antiviral.2010.06.004 20561543

[B18] Liu M Chen W Ni Z Yan W Fei L Jiao Y Zhang J Du Q Wei X Chen J Liu Y Zheng Z Cross-inhibition to heterologous foot-and-mouth disease virus infection induced by RNA interference targeting the conserved regions of viral genome Virology 2005 336 51 59 10.1016/j.virol.2005.01.051 15866070

[B19] Fahim M Ayala-Navarrete L Millar AA Larkin PJ Hairpin RNA derived from viral NIa gene confers immunity to wheat streak mosaic virus infection in transgenic wheat plants Plant Biotechnol J 2010 8 821 834 10.1111/j.1467-7652.2010.00513.x 20374525

[B20] Kanginakudru S Royer C Edupalli SV Jalabert A Mauchamp B Chandrashekaraiah Prasad SV Chavancy G Couble P Nagaraju J Targeting ie-1 gene by RNAi induces baculoviral resistance in lepidopteran cell lines and in transgenic silkworms Insect Mol Biol 2007 16 635 644 10.1111/j.1365-2583.2007.00753.x 17894559

[B21] Golding MC Long CR Carmell MA Hannon GJ Westhusin ME Suppression of prion protein in livestock by RNA interference Proc Natl Acad Sci USA 2006 103 5285 5290 10.1073/pnas.0600813103 16567624 PMC1459347

[B22] Wang PY Jiang JJ Li N Sheng JL Ren Y Chen CF Guo ZR Transgenic mouse model integrating siRNA targeting the foot and mouth disease virus Antiviral Res 2010 87 265 268 10.1016/j.antiviral.2010.02.319 20176056

[B23] Ding S Wu XH Li G Han M Zhuang Y Xu T Efficient transposition of the piggyBac resource (PB) transposon in mammalian cells and mice Cell 2005 122 473 483 10.1016/j.cell.2005.07.013 16096065

[B24] Chen WZ Yan WY Du QY Fei LA Liu MQ Ni Z Sheng ZT Zheng ZX RNA interference targeting VP1 inhibits foot-and-mouth disease virus replication in BHK-21 cells and suckling mice J Virol 2004 78 6900 6907 10.1128/JVI.78.13.6900-6907.2004 15194766 PMC421660

[B25] Reed LJ Muench HA A simple method of estimating fifty percent end points Am J Hyg 1938 27 493 497

[B26] Hasuwa H Kaseda K Einarsdottir T Okabe M Small interfering RNA and gene silencing in transgenic mice and rats FEBS Lett 2002 532 227 230 10.1016/S0014-5793(02)03680-3 12459495

[B27] Rubinson DA Dillon CP Kwiatkowski AV Sievers C Yang L Kopinja J Zhang M McManus MT Gertler FB Scott ML Van Parijs L A lentivirus-based system to functionally silence genes in primary mammalian cells, stem cells and transgenic mice by RNA interference Nat Genet 2003 33 401 406 10.1038/ng1117 12590264

[B28] Tiscornia G Singer O Ikawa M Verma IM A general method for gene knockdown in mice by using lentiviral vectors expressing small interfering RNA Proc Natl Acad Sci US 2003 100 1844 1848 10.1073/pnas.0437912100 PMC149921 12552109

[B29] Wang H Wu J Liu X He H Ding F Yang H Cheng L Liu W Zhong J Dai Y Li G He C Yu L Li J Identification of short hairpin RNA targeting foot-and-mouth disease virus with transgenic bovine fetal epithelium cells PLoS One 2012 7 e42356 10.1371/journal.pone.0042356 22905125 PMC3414509

[B30] Ter Brake O Konstantinova P Ceylan M Berkhout B Silencing of HIV-1 with RNA interference: a multiple shRNA approach Mol Ther 2006 14 883 892 10.1016/j.ymthe.2006.07.007 16959541

[B31] Grubman MJ Baxt B Foot-and-mouth disease Clinl Microbiol Rev 2004 17 465 493 10.1128/CMR.17.2.465-493.2004 PMC387408 15084510

[B32] O‘Reilly EK Kao CC Analysis of RNA-dependent RNA polymerase structure and function as guided by known polymerase structures and computer predictions of secondary structure Virology 1998 252 287 303 10.1006/viro.1998.9463 9878607

[B33] Lu SH Cullen BR Adenovirus VA1 noncoding RNA can inhibit small interfering RNA and microRNA biogenesis J Virol 2004 78 12868 12876 10.1128/JVI.78.23.12868-12876.2004 15542639 PMC524998

[B34] Andersson MG Haasnoot PCJ Xu N Berenjian S Berkhout B Akusjarvi G Suppression of RNA interference by adenovirus virus-associated RNA J Virol 2005 79 9556 9565 10.1128/JVI.79.15.9556-9565.2005 16014917 PMC1181602

[B35] Cheng G Chen W Li Z Yan W Zhao X Xie J Liu M Zhang H Zhong Y Zheng Z Characterization of the porcine alpha interferon multigene family Gene 2006 382 28 38 16901658 10.1016/j.gene.2006.06.013

[B36] Karpen GH Position-effect variegation and the new biology of heterochromatin Curr Opin Genet Dev 1994 4 281 291 10.1016/S0959-437X(05)80055-3 8032206

[B37] Kong Q Wu M Huan Y Zhang L Liu HY Bou G Luo Y Mu Y Liu Z Transgene expression is associated with copy number and cytomegalovirus promoter methylation in transgenic pigs PLoS One 2009 4 e6679 10.1371/journal.pone.0006679 19688097 PMC2723931

[B38] Bueler H Raeber A Sailer A Fischer M Aguzzi A Weissmann C High prion and PrP^Sc^ levels but delayed-onset of disease in scrapie-inoculated mice heterozygous for a disrupted PrP gene Mol Med 1994 1 19 30 8790598 PMC2229922

[B39] Grosveld F Vanassendelft GB Greaves DR Kollias G Position-independent, high-level expression of the human beta-globin gene in transgenic mice Cell 1987 51 975 985 10.1016/0092-8674(87)90584-8 3690667

[B40] Bian Q Belmont AS BAC TG-EMBED: one-step method for high-level, copy-number-dependent, position-independent transgene expression Nucleic Acids Res 2010 38 e127 10.1093/nar/gkq178 20385594 PMC2887973

